# Lessons Learned from the COVID-19 Pandemic: A Survey-Based Study on a Sample of Italian Physicians’ Opinions on Telemedicine

**DOI:** 10.3390/jpm13081267

**Published:** 2023-08-16

**Authors:** Pamela Tozzo, Arianna Delicati, Beatrice Marcante, Dolores Catelan, Luciana Caenazzo

**Affiliations:** 1 Legal Medicine Unit, Department of Cardiac, Thoracic, Vascular Sciences and Public Health, University of Padova, 35121 Padova, Italy; arianna.delicati@phd.unipd.it (A.D.); beatrice.marcante@unipd.it (B.M.); luciana.caenazzo@unipd.it (L.C.); 2 Unit of Biostatistics, Epidemiology and Public Health, Department of Cardiac, Thoracic, Vascular Sciences and Public Health, University of Padova, 35121 Padova, Italy; dolores.catelan@ubep.unipd.it

**Keywords:** telemedicine, telehealth, medical ethics, questionnaire, survey

## Abstract

Telemedicine was born out of the need to ensure clinical evaluation and personal care regardless of the physical presence of the healthcare professional nearby. Information technologies have been vital during the COVID-19 pandemic to ensure medical care and avoid the contagion between patients and clinicians. Accordingly, telecare services multiplied worldwide and gained paramount importance. The present work aims to collect field-based opinions about Telemedicine and ethics among Italian physicians. We developed a web-based questionnaire that was administered to Italian physicians from 1 May to 15 June 2022. The questionnaire was distributed as a link to Google Forms via social networks/instant messaging applications to groups of graduated and qualified physicians. A total of 180 physicians answered the questionnaire (with an age range from 25 to 68 years old). Physicians belonging to the medical area of expertise appear to more frequently use new technologies in comparison to other specialties. The vast majority believe that it is appropriate to use Telemedicine for monitoring and follow-up but not for evaluating a new patient. Concerns about changes in the physician–patient relationship, informed consent, digital barrier, and privacy and data protection also emerged. Finally, telehealth is thought to be a potential useful tool for the future by the majority of respondents but proper training for physicians is therefore needed.

## 1. Introduction

Telemedicine, and more generally e-health, is one of the sectors with the highest rate of innovation; after pharmaceuticals and medical devices, it is considered one of the most relevant industries in healthcare. “Telemedicine” is a term first adopted in the 1970s and can be defined as a method of providing healthcare service through Information and Communication Technologies (I.C.T.) in situations where health practitioners and patients are not in the same place [1]. Since 1997, the World Health Organization has defined Telemedicine as “the delivery of health care services, where distance is a critical factor, by all health care professionals using information and communication technologies for the exchange of valid information for diagnosis, treatment and prevention of diseases and injuries research and evaluation, and for the continuing education of health care providers, all in the interests of advancing the health of individuals and their communities” [2].

According to Italian Telemedicine guidelines, Telemedicine services are classified into three macro-categories: specialized Telemedicine, tele-healthcare, and tele-assistance [3]. The first category, specialized Telemedicine, includes the ways that remote medical services are provided within a specific medical discipline. Depending on the kind of relationship between the individuals involved (professional and patient), the services of specialized Telemedicine can be achieved in different ways, such as televisit, teleconsultation, or health tele-cooperation. Televisit consists of healthcare providers using modern technology to provide real-time or deferred care to distant patients, both diagnostic or therapeutic (prescribing drugs or treatments). Teleconsultation is an indication of diagnosis or choice of therapy between two or more health professionals without the patient’s physical presence, allowing the physicians to ask for a second opinion from colleagues with more significant expertise in a specific field. Finally, health tele-cooperation is the assistance provided by a physician or other health professional to another colleague, especially in an emergency setting.

Tele-healthcare is the second category, and it refers to all services and systems that connect patients, especially chronic ones, with physicians engaged in diagnosing, monitoring, and management. Tele-healthcare involves using I.C.T. to deliver health care at a distance and support patient self-management through remote monitoring and personalized feedback, which also requires an active role of the patient.

The third category is tele-assistance, which is a socio-assistance system for managing elderly or frail persons at home through alarms, activation of emergency services, or support calls from a service center, and it prevalently has social content in order to guarantee assistance continuity.

In recent years, Telemedicine has been increasingly practiced, providing several benefits in healthcare such as reducing emergency room access and hospitalization times, in particular; a 30–35% reduction in mortality and a 15–20% decrease in hospitalizations [4]; reducing waiting times for specialty consultations; being cost-saving [5,6], for example for the delivery of outpatient pulmonary care to a rural population (USD 335 per patient/year) compared to routine care (USD 585 per patient/year) and on-site care (USD 1166 per patient/year); and overcoming isolation for disadvantaged and underserved populations, for example, people living in remote or rural areas [7], as well as improving access to care for disabled, elderly, or fragile patients with mobility problems and sharing knowledge and experiences between professionals.

Telecare services multiplied worldwide during the last pandemic [8]. On 11 March 2020, COVID-19 was declared a pandemic by the World Health Organization [9], radically changing the way medicine has been practiced. In this time of health emergency, two priorities were crucial: at first, ensuring home care for people affected by COVID-19 and for those who, although not infected, presented the need for home care due to their pathological conditions or frailty; on the other hand, avoiding contagion and protecting healthcare professionals.

COVID-19 rapidly accelerated digital health services worldwide as never before, and thus, the need for implementing Telemedicine technologies has never been more relevant [10]. Alongside the exponential spread of Telemedicine in the last two decades, the scientific community felt the urge to promote the use of new technologies in healthcare consistent with the principles of medical ethics [11].

The leading international organizations and professional societies have been providing several ethical guidelines in this field since the late 1990s. The World Health Organization document “A Health Telematics Policy” (1998) discussed the potential of health telematics in support of democratic and equitable global health development, dealing for the first time with cultural, ethical, and legal issues regarding Telemedicine [2]. The “World Medical Association statement on accountability, responsibilities and ethical guidelines in the practice of telemedicine” adopted in 1999 underlined critical ethical issues in the use of Telemedicine, such as the relationship between physicians and patients, the role of the patient, consent and confidentiality, and quality of care and safety, and gave recommendations in practicing telehealth [12]. This document was then updated in 2007 with the following “World Medical Association statement on ethics of telemedicine” [13]. The European Union’s 2013 “Telehealth Services Code of Practice for Europe” included increasing transparency of mission statements and ethical principles, changing clinicians’ professional roles, including special training, viewing patients as active participants in their healthcare, and ensuring they have enough information to make the consent meaningful [14].

Even national codes of medical ethics were updated considering the rising importance of Telemedicine. For example, in Italy in 2014, the Italian Medical Code of Ethics was implemented with the annex to art.78, entitled “Information technologies”. Similarly, the American Medical Association published the “Telehealth implementation playbook” in 2019 and updated it in 2022 [15].

Ethical issues concerning the practice of Telemedicine are increasingly frequently discussed in the scholarly literature and reviews, including informed consent (information about the risks and benefits of remote therapy), patient autonomy, quality of care, equity in healthcare access, patient–professional relationship privacy, malpractice and professional liability, confidentiality, cybersecurity, and data protection [1,11,16].

Considering the burden of Telemedicine in daily clinical practice and the importance of related ethical implications, the present study aims to collect field-based opinions from a sample of Italian physicians about Telemedicine and ethics among Italian physicians, raising awareness about emerging ethical challenges related to the use of new technologies and digital services in medicine.

## 2. Materials and Methods

Firstly, bibliographic research about the main ethical issues related to Telemedicine practice was performed, highlighting the new challenges that arose during the COVID-19 crisis.

**Figure 1:**
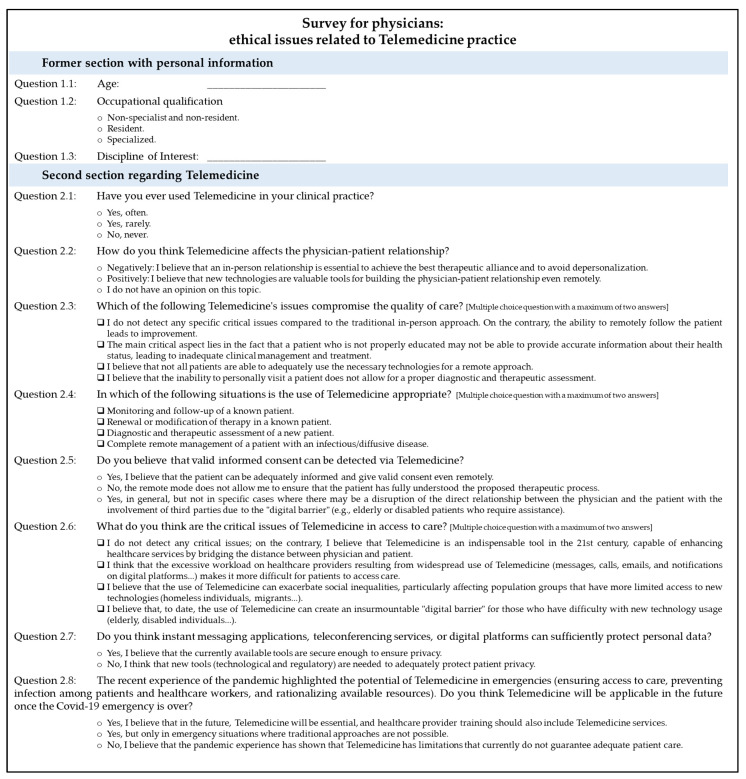
Survey for physicians, English translation.

Based on the literature findings, our team developed a web-based questionnaire. The questionnaire was subsequently tested on a small number (n = 20) of physicians belonging to different professional areas at our institution, reflective of potential respondents. On the basis of their comments and suggestions, the questions were modified, with the help of a biomedical statistical expert. The cross-sectional study was conducted from 1 May to 15 June 2022, comprising senior and junior physicians. A self-designed, pilot-tested, online questionnaire was distributed among all the participants after obtaining informed consent from each of them. The English translation of the questionnaire, originally in Italian, is reported in Figure 1.

The survey was divided into two parts. The former section (Questions 1.1, 1.2, and 1.3) aimed to collect general information such as the responder’s age, occupational qualification (specialist physician, resident, non-specialist, and non-resident physician), and discipline of interest. The second section included eight questions regarding the following topics: the use of Telemedicine in clinical practice and future potentialities of its applications, doctor–patient relationship, quality of care, informed consent, access to care, and data protection. Questions 2.1, 2.2, 2.5, 2.7, and 2.8 were single-answer questions, while Questions 2.3, 2.4, and 2.6 allowed responders to give a maximum of one or two concurrent answers. All questions were mandatory; therefore, submitting an incomplete questionnaire was not possible.

The questionnaire was primarily tested on a small number of physicians (n = 10) of different ages and specializations, reflective of potential respondents. No substantial criticality was reported. Thus, the questionnaire was distributed as a link to Google Forms via social networks/instant messaging applications (Telegram and WhatsApp) to groups of graduated and qualified physicians, such as working chats of different local medical associations or Alumni groups of several Italian universities. The subjects who received the questionnaire via chat or via social networks were in turn invited to spread it to other colleagues with the same communication channels, therefore we are not able to exactly estimate the number of doctors who received the questionnaire.

Study data were collected and managed using REDCap (Research Electronic Data Capture) electronic data capture tools hosted at our institution [17,18]. The analysis was performed using Jamovi software (version 2.3) [19] and STATA software version 16 (StataCorp LP, College Station, TX, USA) [20]. We described data using frequency tables and percentages. We performed a chi-square test of independence and logistic regression to study relations between variables. In particular, we tested the independence between variables with a chi-square test, and if it was rejected, we performed a logistic regression analysis to evaluate the strength of association as Odds Ratio (OR). A type 1 error of 5% was considered for hypothesis testing, and a 95% confidence interval (95% CI) was reported for Odds Ratio (OR). The graphical representations of the results were elaborated with the “RStudio” (Posit Software, PBC formerly RStudio, PBC, Boston, USA) software [21,22].

## 3. Results

**Figure 2:**
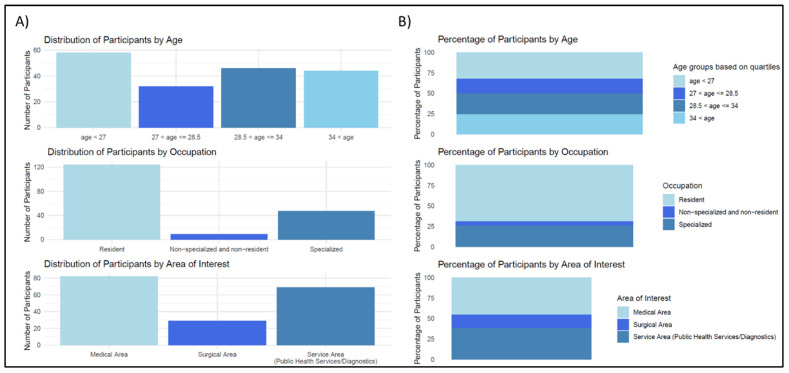
Graphical distribution of participants based on age, occupation, and area of interest. (**A**) Number of participants in the different categories. (**B**) Percentage of participants in the different categories.

A total of 180 physicians answered the questionnaire, and we are not able to estimate the response rate as the subjects who received the questionnaire via chat or via social networks were in turn invited to spread it to other colleagues using the same communication channels. The respondents’ ages range from 25 to 68 years old, with an average age of 34.25 and a mode of 28. The distribution of age groups, occupational classification, and areas of interest is given in Table 1 and in Figure 2.

**Figure 3:**
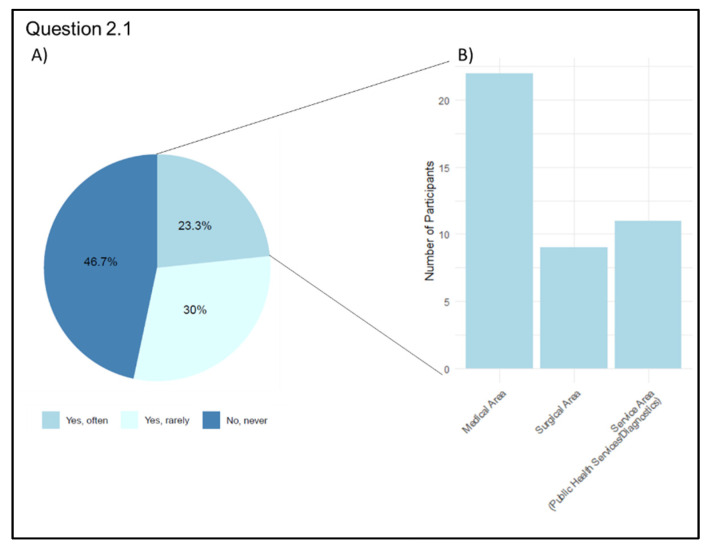
Graphical representation of Telemedicine frequency usage in clinical practice (Question 2.1). (**A**) Participants’ answers: percentage of each category. (**B**) Number of participants who have used Telemedicine frequently grouped by area of interest.

When asked about their personal experience with the use of Telemedicine (Q 2.1 “Have you ever used Telemedicine in your clinical practice?”), 96 physicians (53.3%) answered that they had used it in their medical practice, 54 of them (56.3%) only used it occasionally, and 42 (43.7%) used it frequently. On the contrary, 84 physicians (46.7%) stated that they had never used Telemedicine in their medical practice. According to the distribution by discipline of interest, among the 42 physicians who responded to frequently using Telemedicine, 22 are medical area physicians (52.4%), 9 are surgeons (21.4%), and 11 are doctors from the Public Health Services/Diagnostics category (26.2%) (Figure 3).

**Figure 4:**
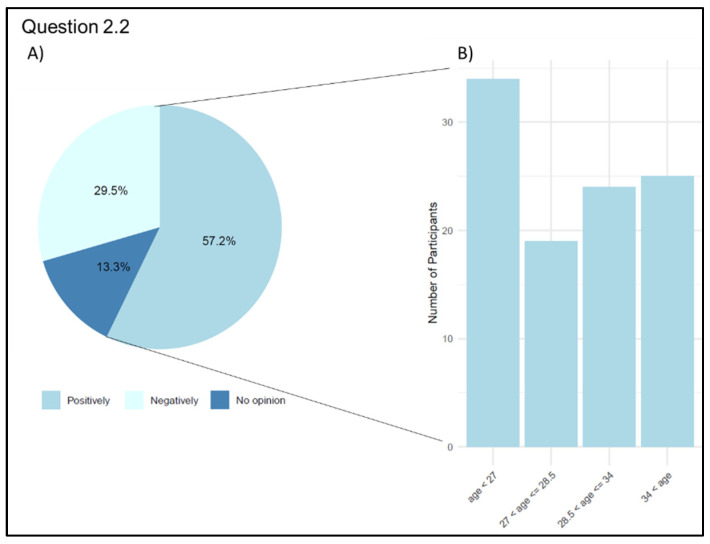
Graphical representation of answers’ frequency on physician–patient relationship (Question 2.2). (**A**) Participants’ answers: percentage of each category. (**B**) Number of participants who responded positively grouped by age on quartiles.

In response to the question about how Telemedicine might change the doctor–patient relationship (Q 2.2: “How do you think Telemedicine affects the physician–patient relationship?”), 103 participants (57.2%) think that Telemedicine may positively affect this connection since technology is perceived as a valuable tool for establishing this relationship, even when the physician and patient are separated. Among the physicians who responded positively, there were 35 physicians (34.0%) under the age of 27, 19 (18.4%) between the ages of 27 and 28.5, 24 (23.3%) between the age of 28.5 and 34, and 25 (24.3%) over the age of 34. For the most remarkable therapeutic alliance and to avoid depersonalization, relation in presence is essential for 53 of our respondents (29.5%); only 24 (13.3%) participants did not have an opinion about this aspect (Figure 4). Regarding the impact of Telemedicine on the physician–patient relationship, from a descriptive perspective, 66.6% of physicians who have already used Telemedicine and 46.4% of those who have never used Telemedicine believe that the new technologies can be valid tools for building the physician–patient relationship even at a distance.

**Figure 5:**
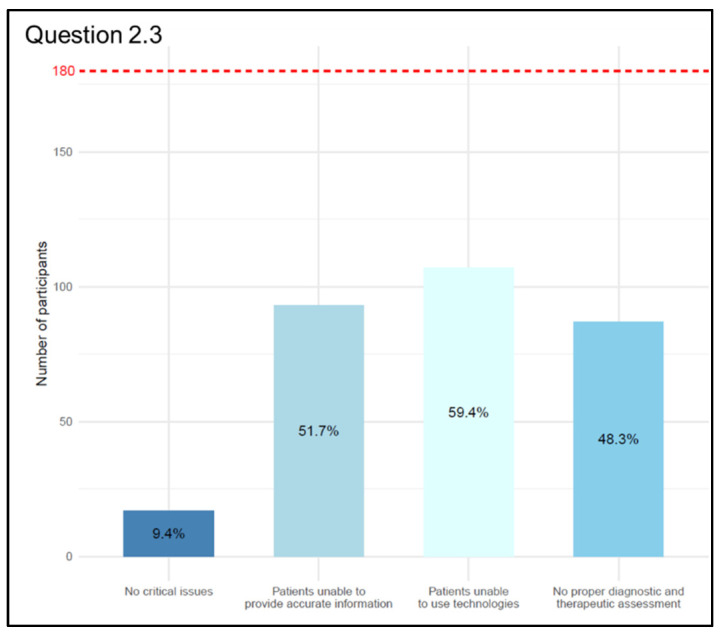
Graphical representation of answers’ frequency percentage on main issues affecting the quality of care compared to the total number of participants (dashed red line) (Question 2.3).

Regarding the main issues affecting the quality of care (Q 2.3 “Which of the following of Telemedicine’s issues compromise the quality of care?” (multiple choice question with a maximum of two answers)), we obtained a total of 304 answers provided by 180 participants, thus some respondents gave more than one answer. The most represented answer underlies that not all patients can adequately utilize the technologies necessary for a remote approach was given by 107 out of 180 participants (59.4%); the answers from 93 participants (51.7%) supported the concept that the criticality lies in the possibility that inappropriately trained patients are unable to provide accurate information about their health status, while 87 participants (48.3%) emphasized the impracticality of an in-person medical visit, making a diagnostic–therapeutic frame impossible. Only 17 participants (9.4%) did not detect any criticality compared to the traditional approach (Figure 5).

**Figure 6:**
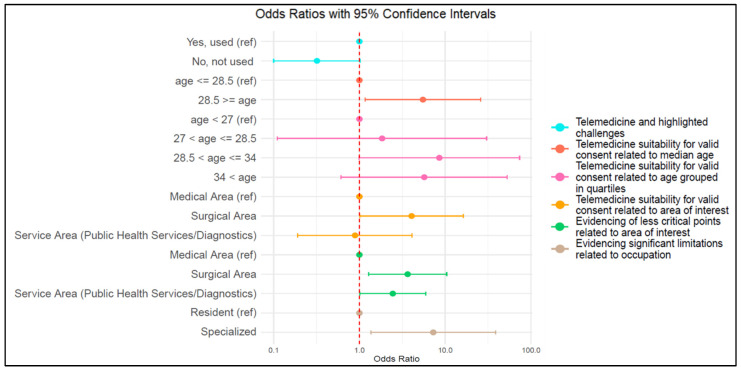
Graphical representation of Odds Ratio and confidence interval as calculated in our sample.

Furthermore, considering those who have already used Telemedicine and those who have not, a significant association (chi-square *p*-value = 0.044) is observed regarding the identification of any challenges related to the quality of patient care. In particular, it was observed that not having used Telemedicine is associated with a lower probability of highlighting the lack of challenges in patient care quality, with an Odds Ratio (OR) of 0.32 (95% CI 0.10; 1.02) (Figure 6). Therefore, those who have never used Telemedicine anticipate a higher number of challenges that are no longer considered as such once this tool was utilized.

**Figure 7:**
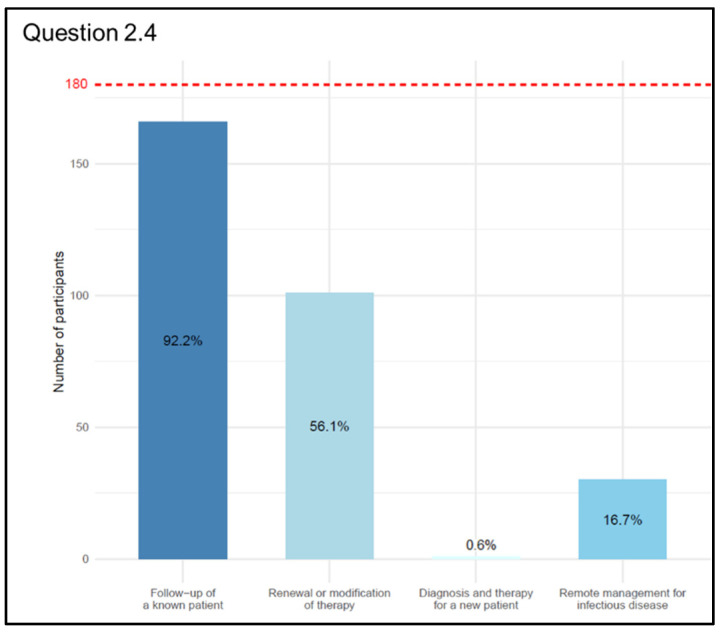
Graphical representation of answers’ frequency percentage of the situations in which the use of Telemedicine is appropriate compared to the total number of participants (dashed red line) (Question 2.4).

Considering the main accepted uses of Telemedicine (Q 2.4 “In which of the following situations is the use of Telemedicine appropriate?” (multiple choice question with a maximum of two answers)), we received a total of 298 responses provided by 180 participants, thus some respondents gave more than one answer. There was a consensus among physicians that deemed the management of known patients as the best use of Telemedicine: 166 physicians answered (92.2%) for the monitoring and follow-up, and 101 out of 180 physicians (56.1%) answered for the renewal or modification of treatments in these situations. On the other hand, for 30 physicians (16.7%), Telemedicine could provide comprehensive remote management in cases of infectious/diffusive diseases, and just 1 (0.6%) answered for the diagnostic–therapeutic framing of a new patient (Figure 7).

**Figure 8:**
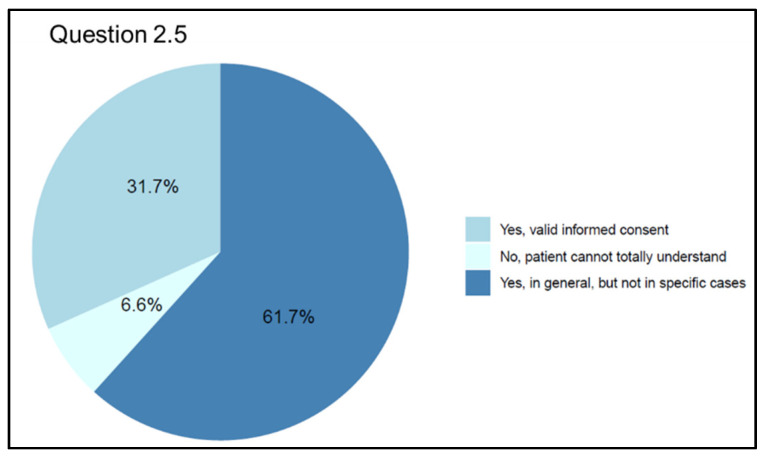
Graphical representation of answers’ frequency percentage on opinions about the possibility to obtain valid informed consent from patients (Question 2.5).

Question 5 (Q 2.5 “Do you believe that valid informed consent can be detected via Telemedicine?”) examined informed consent as a topic. Despite 57 questionnaire participants (31.7%) believing that Telemedicine tools can be used to collect valid informed consent, the majority (n = 111; 61.7%) think that third parties may interfere with the direct relationship between doctor and patient due to the so-called “digital barriers”. Only a small percentage (n = 12; 6.6%) of physicians believe it is impossible to assess patients’ understanding when using Telemedicine (Figure 8).

Our sample revealed a significant association between this variable and the age of the study participants. Specifically, by categorizing the participants into two groups of equal size based on the median age (age ≤ 28.5 years and age > 28.5 years), a greater tendency was observed, as the participants’ age increased, to consider that Telemedicine does not allow for a truly informed consent from the patient, with a chi-square *p*-value of 0.017 and an OR of 5.50 (95% CI 1.17; 25.86) (Figure 6).

This finding was further confirmed by repeating the analysis and categorizing the participants into four age classes based on quartiles (Table 2 and Figure 6). Except for the second class, where there is a “slight increase” with an OR of 1.84, which differs from the first class by at most 1.5 years of age, it is clear that older participants belonging to the third (OR 8.55; 95% CI 0.99; 73.79) and fourth (OR 5.70; 95% CI 0.61; 52.92) categories are more likely to perceive Telemedicine as less suitable for obtaining valid consent from the patient.

The surgeons appear to be more inclined to believe that the telemedical method of obtaining consent does not allow the patient to fully understand the proposed therapeutic process (chi-square *p*-value = 0.044). In fact, as shown in Table 3 and in Figure 6, while participants from the medical and service areas are quite unanimous in stating that the telemedical modality still ensures adequate information for obtaining patient consent, participants from the surgical area are inclined to believe that the consent is not adequate, with an OR four times higher than that of the medical area taken as reference.

**Figure 9:**
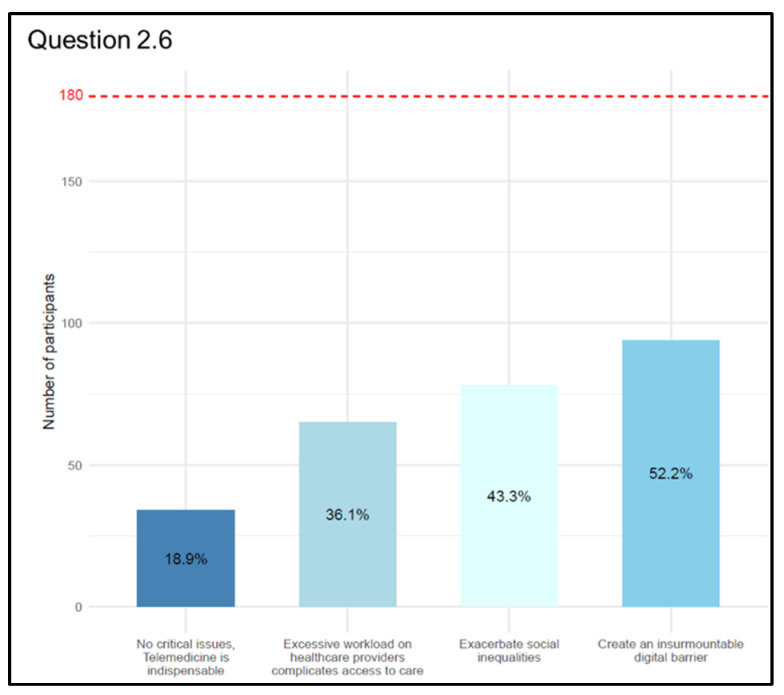
Graphical representation of answers’ frequency percentage on main issues affecting the access to care compared to the total number of participants (dashed red line) (Question 2.6).

Then, we asked physicians how they perceived access to care (Q 2.6 “What do you think are the critical issues of Telemedicine in access to care?” (multiple choice question with a maximum of two answers)), and we received 271 answers. As a result of their responses, 94 replies (52.2%) indicated that Telemedicine might be an insurmountable “digital barrier” for those with have difficulty using new technologies (elderly and disabled) as of today; 78 (43.3%) indicated that the use of Telemedicine may exacerbate social inequalities, which may affect segments of the population who are less likely to access new technologies (homeless people and migrants...); and 65 (36.1%) identified the excessive workload on health care providers resulting from the widespread use of Telemedicine as the main criticality (messages, calls, e-mails, and notifications on computer portals...). Only 34 (18.9%) expressed the absence of criticality in Telemedicine (Figure 9).

With regard to the challenges of Telemedicine in terms of access to care, a significant association (chi-square *p*-value = 0.031) was observed between physicians in the medical area and the other categories of physicians. Specifically, compared to participants in the medical area, the ORs of participants in the surgical and service areas (Table 4 and Figure 6) indicate an increase of 3.65 and 2.45 times, respectively, in their belief that there are no critical points compared to the traditional in-person approach.

**Figure 10:**
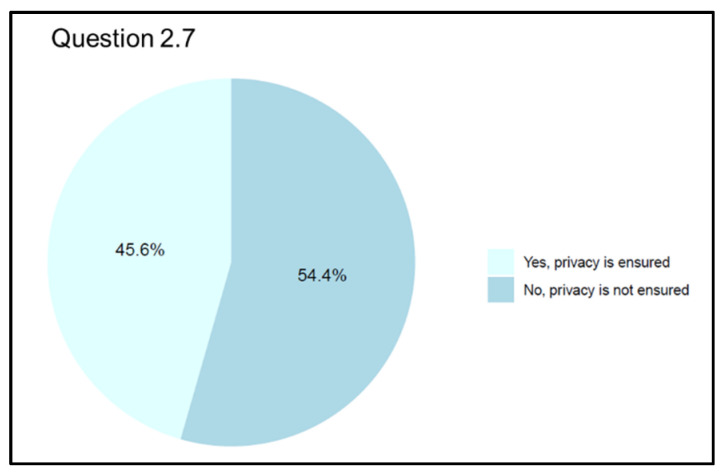
Graphical representation of answers’ frequency percentage on opinions about reliability of data protection (Question 2.7).

Regarding the protection of health data (Q 2.7 “Do you think instant messaging applications, teleconferencing services, or digital platforms can sufficiently protect personal data?”), a slightly higher percentage (n = 98, 54.4%) of physicians who participated in the survey believed that new technological and regulatory tools are unnecessary to ensure adequate privacy protection. As for the other half (n = 82, 45.6%), they believe that the tools available at present are sufficient to protect a patient’s privacy (Figure 10).

**Figure 11:**
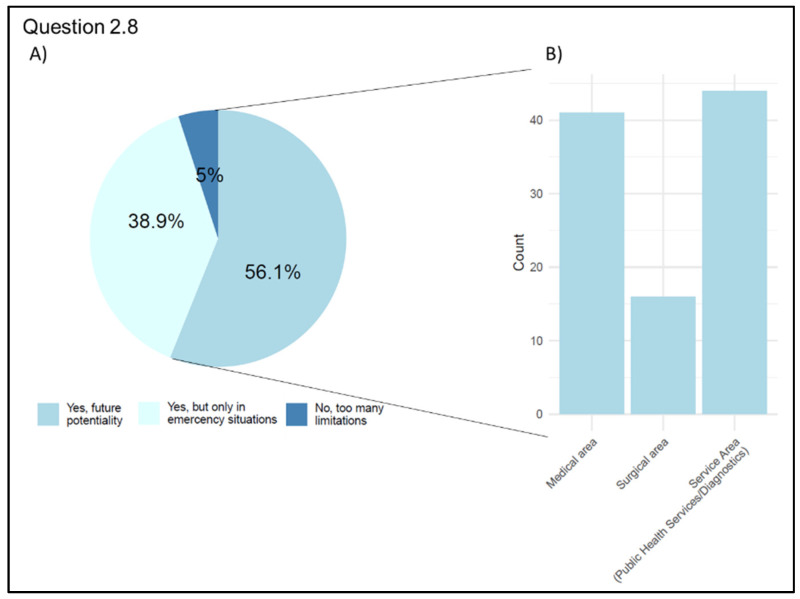
Graphical representation of answers’ frequency on telemedicine potentialities (Question 2.8). (**A**) Participants’ answers: percentage of each category. (**B**) Number of participants who believe that in the future telemedicine will be essential grouped by area of interest.

The last question (Q 2.8 “The recent experience of the pandemic highlighted the potential of Telemedicine in emergencies (ensuring access to care, preventing infection among patients and healthcare workers, and rationalizing available resources). Do you think Telemedicine will be applicable in the future once the COVID-19 emergency is over?”) investigated physicians’ opinions about the applicability of Telemedicine to medical practice in a future perspective, including a post-pandemic scenario. Most participants (n = 101, 56.1%) considered this type of care as beneficial and believe healthcare providers should be trained to use it appropriately in the future. A total of 70 respondents (38.9%) think it should be limited to emergencies. The remaining (n = 9, 5.0%) believe that the recent pandemic experience demonstrated the ineffectiveness of its use in treating patients. Distribution by the area of interest highlighted how the majority of surgeons (n = 16, 55.2%) and physicians from the Public Health Services/Diagnostics category (n = 44, 63.8%) are optimistic about Telemedicine’s future and believe that it will become routine, while the physicians of the medical area are divided on the possibility of considering Telemedicine as indispensable in the future (n = 41, 50.0%) and believe that its future use will be limited to emergency situations where a traditional approach is not possible (n = 37, 45.1%) (Figure 11).

The residents, compared to non-specialists and non-resident and specialized physicians, are the ones who see the greatest future potential of Telemedicine. In fact, 98.4% of them believe that in the future, even after the pandemic, such tools can be utilized. In contrast, 10.6% of specialized physicians and 22.2% of non-specialist and non-resident physicians believe that the pandemic experience has shown how the current limitations of Telemedicine do not ensure adequate patient care. We excluded the category of non-specialist and non-resident physicians due to their limited number. The subsequent analysis was therefore conducted on 171 samples, and a chi-square *p*-value of 0.008 was observed with an OR of 7.26 (95% CI 1.36; 38.84), indicating a 7.26-fold increase in the likelihood of considering Telemedicine characterized by significant limitations among the group of specialized physicians compared to residents (Figure 6).

## 4. Discussion

Telemedicine played a crucial role during the COVID-19 outbreak, proving an essential and life-saving modality for providing healthcare during the pandemic emergency. It is therefore unsurprising that telehealth services expanded and quickly accelerated worldwide, being in most cases a forced alternative to the traditional in-person approach to avoid contagion, protect healthcare professionals, manage infected patients at a distance to relieve the load on hospitals and clinics, follow up on chronic and fragile patients, and much more. Of no less importance, Telemedicine allowed the saving of costs and rationalization of resources during the concurring economic crisis triggered by the pandemic.

On the other side, during an emergency, there is not enough time to delve into ethical or social issues beyond the immediate need [11]. It is well-known that the complexities of remote care delivery, monitoring, and patient–provider communication can lead to unintended consequences of telehealth usage [23], which became even more evident during the pandemic.

In addition, previous works showed apparent differences in priorities between published Telemedicine ethical guidelines and practitioners’ perspectives [24]. In particular, the guidelines seem to primarily focus on macro dimensions and structural aspects of Telemedicine, while practitioners’ concerns are about applying guidelines to specific micro-level contexts and behavioral challenges. Development of new guidelines, updated to the most recent achievements of Telemedicine during the pandemic, should take more into account the practitioner’s perspectives. This paper was intended to collect field-based opinions from healthcare practitioners during the pandemic, and noticeably 53.3% of our respondents affirmed that they have already used, rarely or frequently, Telemedicine. The use of telemedicine, with reference to the physicians’ perspectives, can raise ethical issues with respect to the topics of the quality of care, the physician–patient relationship, the patient’s consent and autonomy, the access and usability of new digital tools and privacy, and confidentiality and cybersecurity.

According to the World Medical Association Statement on the Ethics of Telemedicine, “face-to-face consultation between physician and patient remains the gold standard of clinical care” [13], so the situation in which it is possible to replace the traditional in-person approach with the telematics approach is a fundamental question in order to maintain the highest standard of care. Data from our survey indicate that 53.3% use Telemedicine, 92.2% think that the best field of use for Telemedicine is the remote monitoring and/or follow-up of known patients, and 59.4% think that the greatest criticality in the application of Telemedicine is that not all patients are able to adequately use the technologies necessary for a remote approach. Even if the administration of the questionnaire through digital platforms may have created a selection bias, the objective of the study was precisely to involve doctors already accustomed to the use of digital tools and therefore potentially more involved in Telemedicine activities. Furthermore, from the analysis of the data, it was observed that depending on the area of practice of the interviewed physicians (medical, surgical, and service areas), there are different challenges of Telemedicine highlighted from the perspective of quality of care. For physicians in the medical area, the main challenges are the inability to conduct an in-person examination and the difficulty for some patients to use the necessary technologies for remote approaches. For surgeons, on the other hand, the main challenge arises from the fact that the inadequately informed patient may not be able to provide accurate information about their health status, thus compromising appropriate clinical and therapeutic management. For physicians working in the service area, the main challenges concern the conditions under which patients interact with Telemedicine tools, such as difficulties in accessing technologies and providing adequate information about their health status through telecommunication means. With regard to the challenges of Telemedicine in terms of access to care, a significant association was observed between specialists in the medical area and the other categories of doctors. This is likely due to the fact that physicians in the medical area have used telemedicine during the pandemic for the follow-up of patients with chronic diseases, predominantly older patients, thus having direct experience of the difficulties some categories of patients face in using these technologies.

In particular, physicians of the medical area most frequently use Telemedicine tools. Following the main international guidelines, the vast majority believe that it is appropriate to use Telemedicine for monitoring and follow-up of a known patient and, sometimes, for modifying the therapy in progress but not for evaluating a new patient. Medicine is changing its face and digital medicine is already part of the present. Digital medicine can be a promising opportunity to increase the efficiency and quality of healthcare while helping to reduce or keep costs under control. According to the physicians interviewed in our survey, the main critical issues are insufficient patient education in the use of new technologies and the inability of the patient to understand and convey information about his/her health status to the physician, and 52.2% of our samples believe that today the use of Telemedicine can constitute an insurmountable digital barrier for those who have difficulty using new technologies. The patient’s education in the correct and autonomous use of these new digital tools is therefore crucial to increase the patient’s active participation in the care pathway. The best for the near future will therefore be to define treatment paths with a high level of customization in which, from time to time, a certain service can still be offered in person in the traditional way but can also be available digitally. In fact, some activities, such as monitoring clinical conditions at home, can be performed exclusively through digital tools, making it possible to fill, especially in chronic patients, an information gap that often complicates the treatment process itself.

The physician–patient relationship needs to evolve in parallel with the development of new digital services. Facing the challenges posed by Telemedicine and by the digitization of healthcare, we know we have to deal with two sets of problems: on the one hand, the ones strictly related to the efficacy of patient care, and on the other, the ones related the bureaucratic aspects of daily clinical practice that may undermine the authenticity of the physician–patient relationship. In particular, in clinical practice, we have to consider that the physician is responsible for deciding in which situations televisit can be used in favor of the patient, but during the televisit, the possibility of exchanging real clinical data, medical reports, images, audio, and video in time should always be guaranteed. According to our study group, regardless of age and work experience, Telemedicine is thought to positively influence the physician–patient relationship for 57.2% of respondents, as long as the physician checks the patient’s competence and ensures that he/she can use the proposed tools profitably, gives correct and complete indurations, and involves the patient in tailored treatment planning. At the same time, 51.7% of the physicians interviewed believe that the main criticality of Telemedicine lies in the fact that the patient who is not properly trained is unable to provide the physician with correct information on his or her state of health in order to guarantee adequate clinical therapeutic management. In our sample, regarding the impact of Telemedicine on the physician–patient relationship, from a descriptive perspective, 66.6% of physicians who have already used Telemedicine and 46.4% of those who have never used Telemedicine believe that the new technologies can be valid tools for building the physician–patient relationship even at a distance. Furthermore, considering those who have already used Telemedicine and those who have not, a significant association has been observed regarding the identification of challenges related to the quality of patient care. In particular, it was observed that not having used telemedicine is associated with a lower probability of highlighting the lack of challenges in patient care quality. Therefore, those who have never used Telemedicine anticipate a higher number of challenges that are no longer considered as such once this tool was utilized. Physicians in certain specialties may be more inclined to embrace new technologies due to their nature of work. For example, radiologists, who frequently use high-tech equipment, may be more comfortable with Telemedicine than other professionals who traditionally rely more on face-to-face interactions.

The activation of telemedicine tools, like any other health treatment, requires the prior adhesion of the patient who should undoubtfully be preceded by adequate information so that the patient is aware and well informed on the methods of the visit, advantages, risks, and protection of their personal data. According to different international guidelines on Telemedicine usage [12,13,14,15], patients should be informed about the distinctive features and potential limitations of Telemedicine services as well as about medical issues and treatment options. A small proportion of our respondents (6.6%) are skeptical about the validity of the consent obtained online. According to our respondents, 31.7% think that the patient can be adequately informed and express valid consent even remotely. Specifically, considering the age of respondents, a greater tendency was observed, as the participants’ age increased, to consider that Telemedicine does not allow for truly informed consent from the patient. While participants from the medical and service areas are quite unanimous in stating that the telemedical modality still ensures adequate information for obtaining patient consent, participants from the surgical area are inclined to believe that the consent is not adequate. Our data also show that the digital barrier appears to be the main critical issue regarding the expression of free and informed patient consent, especially in those cases, for example, concerning old or disabled patients, where there may be the interposition of people beyond the patient (family, caregiver, or others). Even when the physician–patient relationship is based on the in-person approach, someone else could influence patient consent, but this issue is obviously exacerbated during a Telemedicine session. The availability of new and fascinating technologies often distracts from the approach to the patient’s health problems, which must be carefully analyzed to find the best application in the right place and at the right time. This analysis concerns the patient’s relationships with everyone involved in the care pathway, including family members and other caregivers, and when it is performed accurately, it will be clear under which circumstances some digital solutions may be acceptable and whether these will represent an advantage for patient care.

Any innovation does not in itself correspond to a positive change, neither for social systems nor for individuals. In the literature, growing importance has been given to the critical issues inherent in the digital barrier and digital divide in the use of Telemedicine services [1,11]. In addition, there has been much discussion about whether the new technologies, while guaranteeing democratization in access to care and providing the ability to reach patients even in remote areas with poor logistical services, may further exacerbate social inequalities, especially to the detriment of the elderly, disabled, poor, and minorities [25]. For these reasons, we should never tire of asking ourselves questions about the characteristics of technologies, the ways in which they are implemented, and the results that their introduction will determine. Patients, who are largely non-digitally literate, should be trained in the correct use of the technology, for example, to know the risk of false positives and consequent false alarms. It should also be avoided that assisted persons can place excessive trust in self-monitoring and in “do-it-yourself” diagnoses, which are not very reliable and cannot be deduced simply from data analysis and without interpretation. A collaboration between physicians and digital tool developers is therefore desirable to integrate the possibilities of technology with the experience of practice, to respond to citizens’ needs in terms of care and assistance. So, telehealth improves access for patients who otherwise would not be getting care, but whether access is sufficiently widely available remains a matter of social justice. Most of the physicians we interviewed noted that the digital barrier is an insurmountable obstacle for those disadvantaged in using new technologies, exacerbating social inequalities. Thus, it may become a further obligation for the physician to identify which patients are genuinely autonomous in the use of new technologies, whether there is a suitable “digital mediator” among their caregivers who can assist the patient in telemedicine practices, or whether the patient can benefit exclusively from a traditional in-person approach. We are therefore faced with the extraordinary opportunity to integrate digital innovation in the best assistance to patients and citizens, protecting the autonomy of the professional and the freedom of choice of the patient, increasing the proximity of the health service towards of citizens and, above all, to increase the trust of the latter in the healthcare system [26]. This scenario can be very interesting due to the strong orientation towards prevention, which is naturally favored by remote assistance and is more easily programmable than in-person assistance, and the possibility to provide specialist services at a distance to the full advantage of the standardization of care.

Compared to the critical issues highlighted by our respondents, some mitigation strategies could be as follows:

- To implement digital education policies of the population, especially of the groups affected by chronic pathologies which could be more involved by telemedicine strategies;
- To implement digital skills of physicians as well so that they too can contribute to patient education; to improve the access of vulnerable groups to technologies they do not have due to economic and/or social problems;
- When enrolling new patients, to use platforms and connection methods that allow adequate timing for in-depth knowledge of the patient to also verify their capability to give valid informed consent using these technologies;
- To ameliorate privacy protection strategies in the Telemedicine platforms used.

The increase in the prevalence of chronic diseases in the population linked to aging makes it necessary to equip systems that allow for better and more sustainable management of these diseases. Telemedicine allows you to pursue these goals. However, to arrive at an ideal management of patients on a telematic basis, the system still needs to improve on the methods of self-monitoring and transmission of data by the patient to the clinical center, on the archiving and processing of data and on the integrated use of the same platform by multiple specialists. The goal of future models of assistance based on digital resources should be to better guarantee a patient’s care at a lower cost with maximum patient benefit. If the idea is to bring healthcare as close to the patient as possible, we could even use digital technology to develop mobile healthcare facilities to respond flexibly to changing healthcare service needs. As mobile health and other means to access telehealth through multiple devices expand, privacy, cybersecurity, data use, and related end-user agreements are gaining more importance [27]. The amount of data collected with physicians’ electronic devices (mobiles, tablets, and computers) is constantly growing, without secure networks or data encryption methods, increasing the risk of a data breach. The need to improve cybersecurity is even more apparent since the burden of hacker attacks against public and private health networks. Again, during the COVID-19 pandemic, location and contact tracing information collection raised new concerns about patient privacy and data security [28]. Moreover, commercial services and healthcare organizations could collect and then sell data for purposes unrelated to healthcare, and concerns about the use of Big Data and artificial intelligence is growing [29]. Consistent with those above, half of our sample believes that the tools for protecting the patient’s digital data are not appropriate and insufficient. It is, therefore, necessary to develop dedicated platforms with a high level of cybersecurity to protect health data, raise awareness of professionals and patients to these issues, strengthen data protection systems, and adapt the regulatory framework with specific regard to privacy in the health sector.

The main limit of this study is that we have collected a limited number of responses in a relatively short period of time. This has been performed in order to capture impressions in the immediate post-pandemic. It could certainly be interesting to expand the sample in the future, and future work should provide specific recommendations for the content and structure of the training of both physicians and patients to make it effective. Moreover, our goal was not to extend and apply the results to the entire population of Italian physicians but to identify and investigate the opinions of those who might already be settled to the use of “smart” technologies.

## 5. Conclusions

The COVID-19 pandemic caused physicians to switch rapidly from the traditional face-to-face approach to telehealth in more and more settings. Many physicians in our study group, especially in the medical area, routinely and productively use digital services in their daily practice and appear sensitive to the main ethical issues concerning Telemedicine.

The patient has assumed an unprecedented role as an active player in the treatment path and therefore needs assistance and education, just like clinicians, in order to take advantage of all the new ways to obtain care and understand the implications, including the ethical considerations, of using telehealth services. Patient education is a vitally critical component of the treatment via telehealth. Telehealth exists, also referring to WHO, to provide care and education to patients. Instruction and clinical support provide patients with critical disease-related information and empower patients to become more deeply engaged in their care and outcomes.

The general direction is marked, and the pathway is being traced. Sooner or later the devices will become reliable, likely in forms that are not even conceivable at the moment, and will certainly find indications of use, at least in selected patients and contexts. The physician should have a constructively critical approach to using the enormous potential of Telemedicine but also know its limits. Surely medicine can never be only virtual or approachable solely with sensors or algorithms. The hope is that not so much technology changes medicine but that the value system of medicine (equality, real needs, accessibility, and continuity of care...) can modulate technology. This should be true innovation with high added value that is flexible, powerful, and cost saving, oriented towards people’s real needs.

## Figures and Tables

**Table 1:** Descriptive characteristics of the respondents.

Age Groups Based on Quartiles	N.	Percentage
age < 27	58	32.2%
27 < age ≤ 28.5	32	17.8%
28.5 < age ≤ 34	46	25.6%
34 < age	44	24.4%
Total	180	100.0%
Occupation		
Resident	124	68.9%
Non-specialized and non-resident	9	5.0%
Specialized	47	26.1%
Total	180	100.0%
Area of interest		
Medical Area	82	45.6%
Surgical Area	29	16.1%
Service Area (Public Health Services/Diagnostics)	69	38.3%
Total	180	100.0%

**Table 2:** Telemedicine as less suitable tools for obtaining valid consent from patients: opinions based on participants’ age grouped in quartiles. OR = Odds Ratio; CI = confidence interval.

Age Groups Based on Quartiles	OR	95% CI
age < 27	Ref.	
27 < age ≤ 28.5	1.84	0.11, 30.42
28.5 < age ≤ 34	8.55	0.99, 73.79
34 < age	5.70	0.61, 52.92

**Table 3:** Telemedicine as less suitable tools for obtaining valid consent from patients: opinions based on participants’ area of interest. OR= odds ratio; CI= confidence interval.

Area of Interest	OR	95% CI
Medical Area	Ref.	
Surgical Area	4.06	1.01, 16.34
Service Area (Public Health Services/Diagnostics)	0.89	0.19, 4.10

**Table 4:** Telemedicine as less affected by critical points: opinions based on participants’ area of interest. OR= odds ratio; CI= confidence interval.

Area of Interest	OR	95% CI
Medical Area	Ref.	
Surgical Area	3.65	1.28, 10.41
Service Area (Public Health Services/Diagnostics)	2.45	1.01, 5.96

## Data Availability

The entire dataset is available upon request to the corresponding author.
